# Oral History in der Medizin und Narrative Medizin – ein Kommentar zur Frage der Vulnerabilität

**DOI:** 10.1007/s00048-024-00377-2

**Published:** 2024-02-27

**Authors:** Agnès Arp

**Affiliations:** Oral-History-Forschungsstelle, Neuere und Zeitgeschichte und Geschichtsdidaktik, Universität Erfurt, Nordhäuser Str. 63, 99089 Erfurt, Deutschland

In diesem Beitrag wird der Umgang mit ethischen und wissenschaftlichen Anforderungen von qualitativen Interviews mit vulnerablen Gruppen im Allgemeinen und mit aus medizinischen Gründen vulnerablen Personen im Besonderen kommentiert. Vulnerabilität bedeutet *Anfälligkeit, Verletzbarkeit* und lenkt in unserem (deutschen) Kontext die Aufmerksamkeit auf Personen, die angesichts von früheren Erfahrungen oder von Prädispositionen emotional empfindlicher sind und eher psychische Störungen entwickeln können als andere. In der Psychologie wird Vulnerabilität als das Gegenteil von Resilienz betrachtet (vgl. Dorsch 2015).

Vor diesem Hintergrund setzen wir uns mit folgenden Fragen auseinander: Ist die Oral History eine geeignete Methode, um vulnerable Personen zu interviewen? Was sind die methodischen und praxeologischen Herausforderungen dieser Methode? Können wir schließlich behaupten, dass Oral History in der Medizin etwas Besonderes ist und einen tatsächlichen Mehrwert für die medizinische Forschung und Praxis bringt?

Dies führt uns zu der Frage, ob die Oral Historians, Sozialwissenschaftler:innen, Historiker:innen ein besonderes psychologisches Vorwissen brauchen, wenn sie lebensgeschichtliche Interviews mit vulnerablen Personen führen. Diese Frage ist natürlich rein rhetorisch: Selbstverständlich kommt es auf die eigene Haltung und das Beherrschen der Oral-History-Methode an, die viel Zeit in der inhaltlichen, themenfokussierten Vorbereitung, in der methodischen Durchführung und in der wissenschaftlichen Nachbereitung fordert.

## Warum ist Oral History eine lohnenswerte Methode?

Oral History geht ursprünglich von den mündlichen Traditionen marginalisierter Gruppen in der Gesellschaft aus, die ihre Geschichte, Kultur und Sprache mündlich an die nächste Generation weitergegeben haben. Die Methoden der akademisierten Oral History in Deutschland zielen in Anlehnung daran auf die Geschichte von Gruppen ab, die in klassischen historischen Quellen in der Regel zu einseitig dargestellt werden und/oder im dominanten historischen Diskurs unsichtbar oder im Hintergrund geblieben sind.

In der deutschen beziehungsweise deutschsprachigen Geschichtswissenschaft hat sich mit der Oral History im Verlauf von etwa 50 Jahren ein eigenes Forschungsfeld herausgebildet, das über Gespräche mit Zeitgenoss:innen Bereiche der Vergangenheit, die über schriftliche Quellen nicht zugänglich sind, erschließt. Oral Historians arbeiten mit Personen, die sie anhören und befragen – gewöhnlich als Interviewpartner:innen bezeichnet – und erzeugen somit ihre mündlichen Quellen. Oral Historians sammeln ihre Erkenntnisse zu bestimmten Themen oder Ereignissen während eines bestimmten Zeitraums und versuchen, die Essenz der Alltagserfahrungen einzufangen, die Anderen möglicherweise nicht bekannt sind oder von ihnen nicht aufgezeichnet werden.

Oral History ist interdisziplinär, da ihre Ursprünge und kritischen Ereignisse eine Reihe von anthropologischen, soziologischen, linguistischen, literarischen und psychologischen Konzepten widerspiegeln. Die Erfahrungen der Menschen als Datenquelle ins Visier zu nehmen, verleiht schließlich der wissenschaftlichen Reichweite der Geschichte neue Breite und Tiefe.

Das Hauptmerkmal mündlicher Quellen ist das Ausmaß der Subjektivität des/der Erzählers/:in. Subjektivität lässt sich dabei für die Geschichte als genauso wichtig verstehen wie Faktengeschichte. Was die Erzähler:innen glauben, kann aus ihrer Sicht als Tatsache betrachtet werden, denn Menschen handeln nach ihren Überzeugungen. Das ist es, was Oral History ausmacht, da wir hauptsächlich dadurch die Bedeutung von Ereignissen für Menschen in ihrem täglichen Leben erst fassen können. Narrative Erinnerungsinterviews (Niethammer 1985) sind eine große Hilfe, weil sie Aspekte beleuchten, die schriftliche Quellen im Schatten belassen haben – zum Beispiel Verbindungen zwischen Menschen, informelle Situationen oder persönliche Aspekte der Biografie einer Person. Aus diesem Grund ist es wichtig, ihre Verwendung zu fördern und neue Historiker:innen in der Methode auszubilden, damit die Erkenntnisse in die aktuellen Berufspraktiken von Archivar:innen und Forscher:innen eingehen, denn die sogenannte Objektivität der schriftlichen Quellen wird zunehmend relativiert.^1^ Oral History kann nicht improvisiert werden. Es gibt nicht nur Regeln, die bei Form und Praxis im Kontext von Interviews zu befolgen sind, sondern auch eine (selbst-)kritische Haltung des/der Interviewers/:in ist Voraussetzung. DasGupta hat dies sehr pointiert formuliert:

„Oral history recognizes the interview as a unique *event *that can neither be reproduced at a different time or with a different interviewer. The uniqueness of the interview event is determined by the relationship of interviewer to interviewee, the nature of the questions asked, and perhaps, as esoteric factors as the time of day, directly prior occurrences, and cues of the physical environment“ (DasGupta 2008).

Darüber hinaus setzt die Oral History eine Fähigkeit zur Selbstreflexion des/der Forschers/:in/Interviewers/:in voraus, der/die sich seiner/ihrer Grenzen und Möglichkeiten bewusst ist und gegebenenfalls seine/ihre Feldarbeit extern supervidieren lässt (siehe u. a. Osterhaus 2011). Eine Fähigkeit, die unmittelbar an die Kompetenz des *aktiven Zuhörens *anknüpft (Miller 2020). Dabei geht es darum, das Bewusstsein für *Kommunikationssperren*,^2^ wie Miller sie nennt, zu verfeinern, um *Aussagen im Sinne reflektierenden Zuhörens zu formulieren *(ebd., Kap. 9).

Die Durchführung von tiefgreifenden Gesprächen und deren qualitative Auswertung sind für die Oral History ein zentraler Forschungsansatz, der sich der Kraft und zugleich Fragilität der Erinnerungen stellt. Die Erzählung setzt die Erinnerung voraus. Sehr ähnlich verhält es sich in der Medizin, wo die Heilungserfolge grundsätzlich von einer differenzierten, genauen Diagnose herrühren.

## Die Erzählung und deren Deutung in der Medizin

Jüngere medizinische Versorgungsansätze greifen das vertrauensvolle Gespräch in der Anamnese als Schlüssel auf, die Patient:innen ernster zu nehmen und in der therapeutischen Entscheidungsfindung zu berücksichtigen (Gusy & Drewes 2012). In diesem Zusammenhang stellt die (selbst-)kritische Haltung im medizinischen Bereich im Kontakt mit Patient:innen eine Dimension dar, die seit ein paar Jahren von der narrativen Medizin aufgegriffen wird. Narrative Medizin^3^ zielt darauf ab, einen wesentlichen Platz der Krankheitserzählung in der Praxis der Medizin wiederherzustellen, indem sie sowohl das Zeugnis der Erzählung der Patient:innen würdigt als auch eine Form der Selbstschreibung und -reflexion bei dem Arzt/der Ärztin. Es geht also darum, dass der Arzt/die Ärztin eine narrative Fähigkeit entwickelt, damit sie/er für die Erzählung des/:r Patienten/:in empfänglich und in der Lage ist, sie zu analysieren. Rita Charon definiert sie so: „Der Begriff ‚narrative Medizin‘ kam mir als synthetischer Begriff für eine bewusste und kompetente medizinische Praxis in der Theorie und Praxis des Lesens, Schreibens, Erzählens und Empfangens von Geschichte“ (Charon 2015: 14). Sie wurde sich der Tatsache bewusst, dass „die Analyse von Erzählungen in gewisser Weise die Praxis der Medizin verändern könnte“ (Charon 2017a: 77). Gemeinsam mit Forschenden aus verschiedenen Disziplinen wird sie über die fürsorgliche Beziehung „zwischen aufmerksamem Lesen und aufmerksamem Zuhören, zwischen Aufmerksamkeit und Repräsentation und wie Aufmerksamkeit und Repräsentation zu Verbundenheit führen“ nachdenken (Charon 2017a: 79). Die erzählerische Kompetenz basiert somit auf einem Dreiklang: Aufmerksamkeit, Repräsentation und Verbundenheit. Schließlich führt die Kombination – nach vertiefenden Übungen – zur aktiven und einfühlsamen Zusammenarbeit zwischen dem Pflegepersonal und dem/:r Patienten/:in. Durch die Anwendung des Dreiklangs von Aufmerksamkeit, Repräsentation und Verbundenheit geht es tatsächlich darum, dem Zeugnis der Kranken „Tribut zu zollen“ (Charon 2015: 21). Der Ausdruck „Tribut zollen“ ist dabei wesentlich, weil er andererseits die Bedeutung dieser Erzählungen insofern unterstreicht, als sie dazu beitragen, die übliche Asymmetrie der medizinischen Macht umzukehren. Daher unterstützt die Würdigung des Zeugnisses der Kranken nicht nur den Zweck der narrativen Medizin, sondern bildet auch durch das Reflexionspotenzial, das in einem bewussten Kommunikationsrahmen vorhanden ist, ein Instrument der Selbstfürsorge der Ärztin/des Arztes.

Der Bedarf nach einer strukturierten Vermittlung gesprächsbasierter Forschungskompetenzen ist fach- und disziplinübergreifend groß. Erzählerische Kompetenz ist der Methode der Oral History in der Interviewführung intrinsisch. Bei schwierigen, hoch emotionalen Themen könnte man behaupten, dass die narrative Methode die Methode der Wahl ist, denn gesprächsbasierte Wissensgewinnung hat immer mit Menschen in spezifischen Kontexten zu tun: „Oral history interviews are unique in that the interaction of researcher and subject creates the possibility of going beyond the conventional stories […] to reveal experience in less culturally edited form.“ (Perks & Thomson 1998).

In Gesprächen spiegelt sich die sich verändernde diskursive Umgebung zum einen in den besprochenen Inhalten, zum anderen auch im gesamten Gesprächsrahmen wider. Auch wenn die Inhalte dieser Gespräche unterschiedlich sind, müssen Forschende, die mit diesen Methoden arbeiten, Techniken und Qualitätsmerkmale der wissenschaftlichen Gesprächsführung beherrschen. Wichtig ist zudem, Grenzen der forschenden beziehungsweise pädagogischen Gesprächsführung zum Beispiel im Hinblick auf ein Therapie- oder Mediationsgespräch zu erkennen und damit professionell umzugehen.

## Oral History in der Medizin – etwas Besonderes?

Die narrative Medizin und die Oral History messen der Narrativität, der offenen, freien Erzählung eine essenzielle Rolle im Arzt/Ärztin-Patient:in– beziehungsweise Interviewer:in–Interviewten-Verhältnis sowie der Kompetenz des Arztes/der Ärztin beziehungsweise des Oral Historians in der Medizin bei. In diesem Sinne könnte man die Frage umdrehen und behaupten, dass die beste Methode, vulnerable Personen zu interviewen, die Oral-History-Methode sei, auch wenn sie sich durchaus nicht bei jeder Person selbstverständlich anwenden lässt, denkt man zum Beispiel an die methodischen Herausforderungen, die die Zusammenarbeit mit Gehörlosen mit sich bringt.^4^

Wenn es im Fall von Interviews mit vulnerablen Personen aus ethischen Gründen besonders wichtig ist, diese Schritte bewusst durchzugehen, bleibt eine methodisch korrekte Interviewführung insofern die Basis jedes Oral-History-Projekts, als das Ergebnis eine historische Quelle geworden ist, die ausgewertet und archiviert wird. In dieser Hinsicht birgt die narrative Kraft der Oral History ein großes Potenzial, heilende Wirkung zu erzeugen. Der/die Oral Historian zwingt dem/der Erzähler:in seine Erwartungen nicht auf, steuert die Geschichte nicht. Gleichzeitig kann er nicht nur *objektiv* bleiben, sondern reflektiert seine Forschung und seine Deutungen, Voreinnahmen und Glaubenssysteme, weshalb ein gewisses Maß an Transparenz mit dem/der Interviewpartner:in notwendig ist. Eine solche gegenseitige Transparenz ist für den medizinischen Dialog und die medizinische Praxis von grundlegender Bedeutung (vgl. DasGupta 2008).

Die Oral-History-Theorie bereichert unser Verständnis der dialogischen Begegnung als beziehungsbildendes Ereignis und der mündlichen Erzählung als eine gemeinsam geschaffene Geschichte, die sowohl den/die Erzählenden als auch den/die Zuhörenden widerspiegelt. Dies ist auch relevant für die medizinische Anamnese, Diagnostik und Therapie.

Wie Nicole Immler richtig festgestellt hat, ist das Archivieren von Lebensgeschichten inzwischen nicht mehr genug. „Menschen geht es häufig nicht nur darum, ihre Geschichte zu erzählen, sondern sie wollen auch sehen, dass die eigene Geschichte Teil der ‚offiziellen‘ Geschichte wird“.^5^ Indem die Oral History den Interviewten eine Stimme verleiht und diese mit professioneller Langzeitarchivierung auch für die Nachwelt sichert, sind bisher ungehörte Erzählungen imstande, Einfluss auf die Geschichtsschreibung zu nehmen. Das ist nicht von geringer Bedeutung, wenn wir uns mit stigmatisierenden, unbequemen oder vernachlässigten Themen beschäftigen.

## Footnotes

1 Das wird an der Oral-History-Forschungsstelle (OHF) der Universität Erfurt, deren Ziel es ist, eine zertifizierte Lehre der Oral History zu etablieren, angeboten. Im Mittelpunkt der Oral-History-Forschungsstelle steht die „ostdeutsche Erfahrung“. Die Forschungsstelle greift die Spezifika der DDR sowie der Zeit der Transformation auf und diskutiert die Methodik der Oral History in diesem Kontext. Die Forschungsstelle entwickelt und etabliert ein Konzept für einen respektvollen und fairen Dialog, der sich insbesondere auch mit Macht- und Partizipationsfragen auseinandersetzt. Im Internet finden sich nähere Informationen dazu auf den Seiten der Professur für Neuere und Zeitgeschichte und Geschichtsdidaktik.

2 Diese sind: anweisen, warnen, beraten, überzeugen, moralisieren, urteilen, zustimmen, beschämen, analysieren, nachforschen, beruhigen und ablenken (Miller 2020: 30–32).

3 Vgl. Rita Charon, Professorin an der Columbia University School of Medicine. Der Begriff „narrative Medizin“ tauchte erstmals 2001 auf. Die ersten Module der Lehre der narrativen Medizin wurden 2006 an der Columbia University eingerichtet und in Form von Workshops präsentiert – in dem Jahr, in dem Rita Charons Buch die Thematik in *Narrative Medicine: Honoring the Stories of Illness *reflektierte. Im Jahr 2009 eröffnete das Narrative-Medicine-Programm einen Master of Science in Narrative Medicine. Im Jahr 2015 gab es 120 Absolvent:innen. Im Juni 2013 fand in London das erste europäische Symposium zum Thema „Narrative Medicine and the Future of Health Care“ statt.

4 An der OHF legen wir besonderes Augenmerk auf eine Oral History der Sinnesbeeinträchtigungen.

5 Vgl. Immler (2019). Beispiele für Oral-History-Forschung in der Physiotherapie: Mary Tremblay, „Going Back to Civvy Street: A Historical Account of the Impact of the Everest and Jennings Wheelchair for Canadian World War II Veterans with Spinal Cord Injury“ (Disability & Society, 1996). Tremblay verwendete Interviews und Archivmaterial, um zu veranschaulichen, wie Rollstühle es Veteranen (und Zivilist:innen) ermöglichten, in ihre Gemeinden zurückzukehren und Arbeits- und Bildungsmöglichkeiten zu finden. Tremblay beschrieb die Arbeit von Veteranen, um eine Organisation zu schaffen, die sowohl Veteranen als auch Zivilist:innen hilft. Sie wies jedoch darauf hin, dass das allgemeine Problem der baulichen Barrieren erst in den 1950er Jahren erkannt wurde. Siehe ebenfalls die Arbeit von Peter Harper im Bereich Genetik. In Großbritannien hat sich die Oral History Medicine bedeutend entwickelt.
